# Supplementary material for: Genome Variability of Infectious Bronchitis Virus in Mexico: High Lineage Diversity and Recurrent Recombination
Source: Viruses. 2023 Jul 20;15(7):1581. doi: 10.3390/v15071581 (PMC10386725; doi:10.3390/v15071581)
Supplement: Supplementary file 1 [file viruses-15-01581-s001.zip › viruses-2452565-supplementary.pdf]

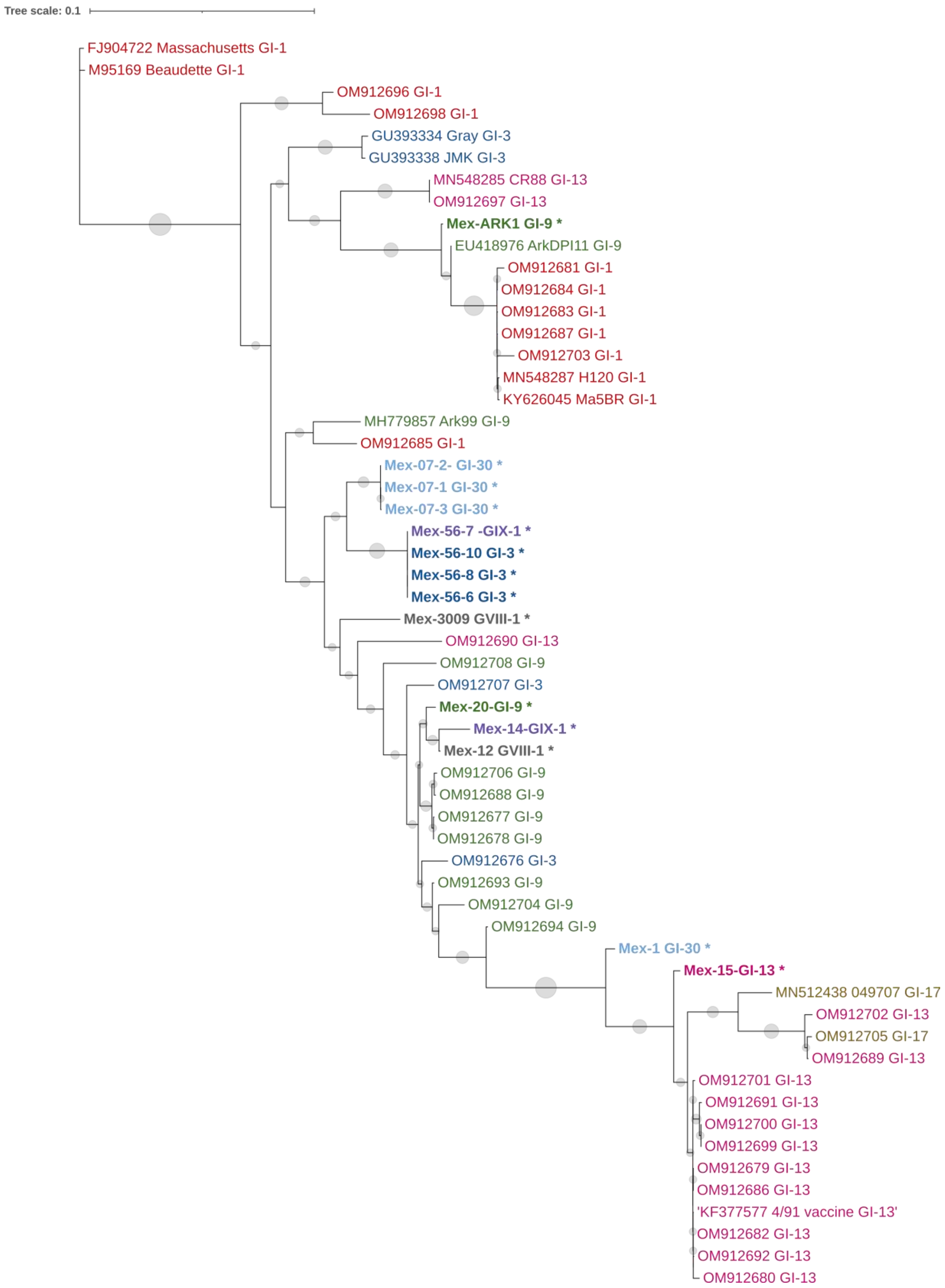

Figure S1. The phylogenetic tree was obtained with the maximum-likelihood method and HKY model with gamma distribution and invariant sites. Phylogenetic reconstruction was carried out using ORF 1a sequences of the genomes here obtained (*n* = 14), other Mexican genomes available (*n* = 33), and reference or vaccine strains reported from Mexico (*n* = 10).

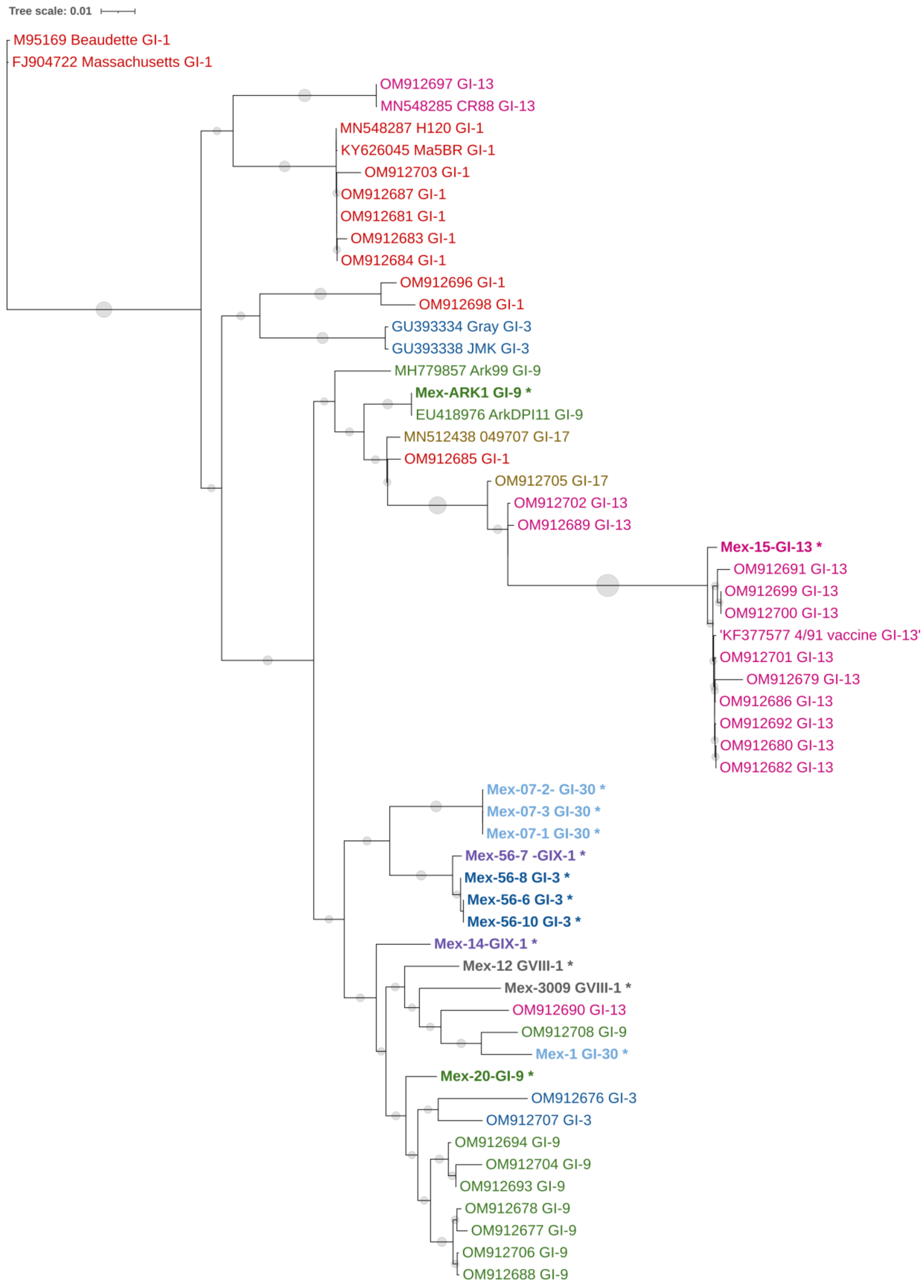

Figure S2. The phylogenetic tree was obtained with the maximum-likelihood method and HKY model with gamma distribution and invariant sites. Phylogenetic reconstruction was carried out using ORF 1b sequences of the genomes here obtained (n = 14), other Mexican genomes available (n = 33), and reference or vaccine strains reported from Mexico (n = 10).

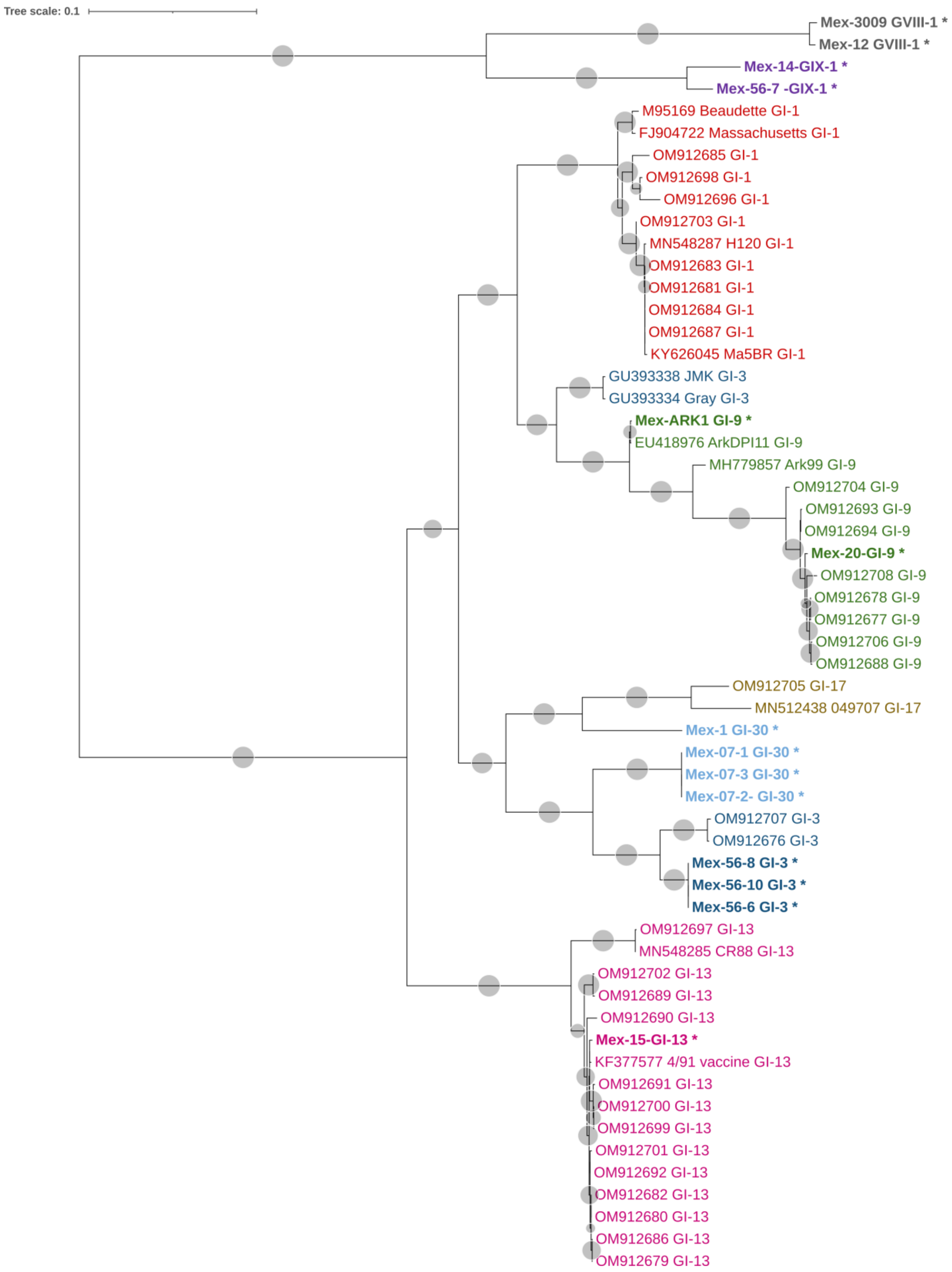

Figure S3. The phylogenetic tree was obtained with the maximum-likelihood method and GTR model with gamma distribution and invariant sites. Phylogenetic reconstruction was carried out using gene 2 sequences of the genomes here obtained (*n* = 14), other Mexican genomes available (*n* = 33), and reference or vaccine strains reported from Mexico (*n* = 10).

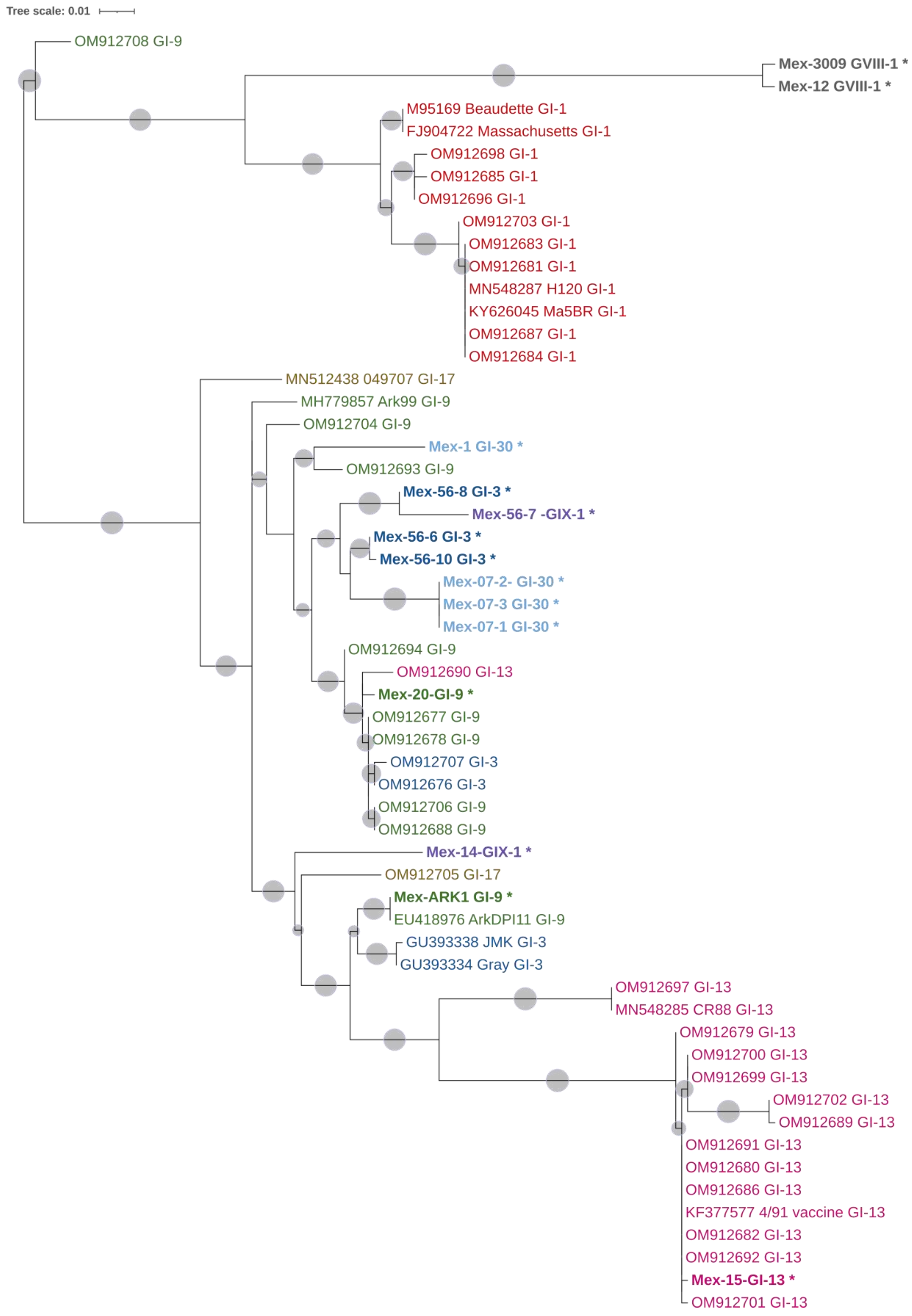

Figure S4. The phylogenetic tree was obtained with the maximum-likelihood method and GTR model with gamma distribution and invariant sites. Phylogenetic reconstruction was carried out using gene 3 sequences of the genomes here obtained (n = 14), other Mexican genomes available (n = 33), and reference or vaccine strains reported from Mexico (n = 10).

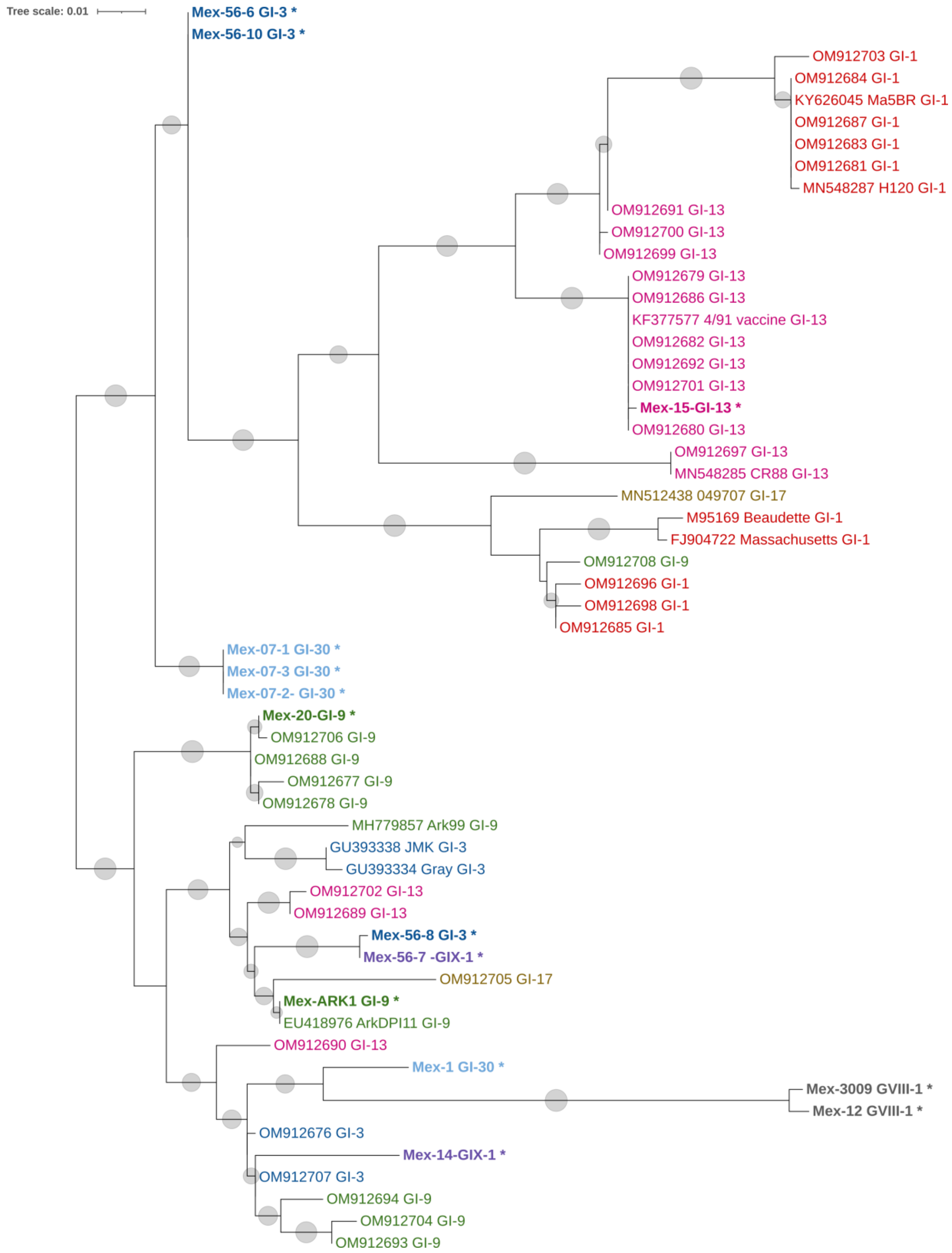

Figure S5. The phylogenetic tree was obtained with the maximum-likelihood method and GTR model with gamma distribution and invariant sites. Phylogenetic reconstruction was carried out using gene 5 sequences of the genomes here obtained ($n = 14$), other Mexican genomes available ($n = 33$), and reference or vaccine strains reported from Mexico ($n = 10$).

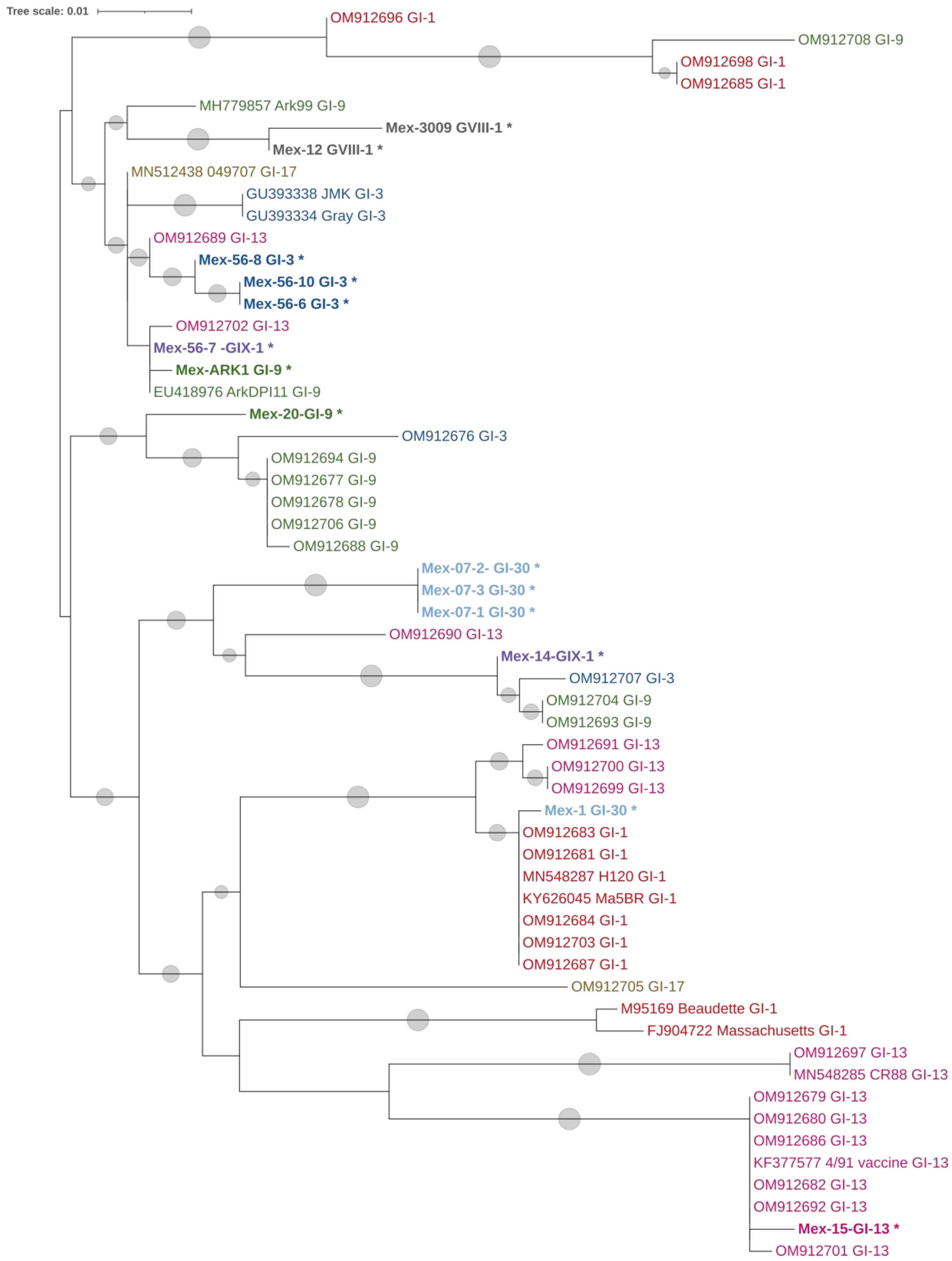

Figure S6. The phylogenetic tree was obtained with the maximum-likelihood method and GTR model with gamma distribution and invariant sites. Phylogenetic reconstruction was carried out using gene 6 sequences of the genomes here obtained (n = 14), other Mexican genomes available (n = 33), and reference or vaccine strains reported from Mexico (n = 10).

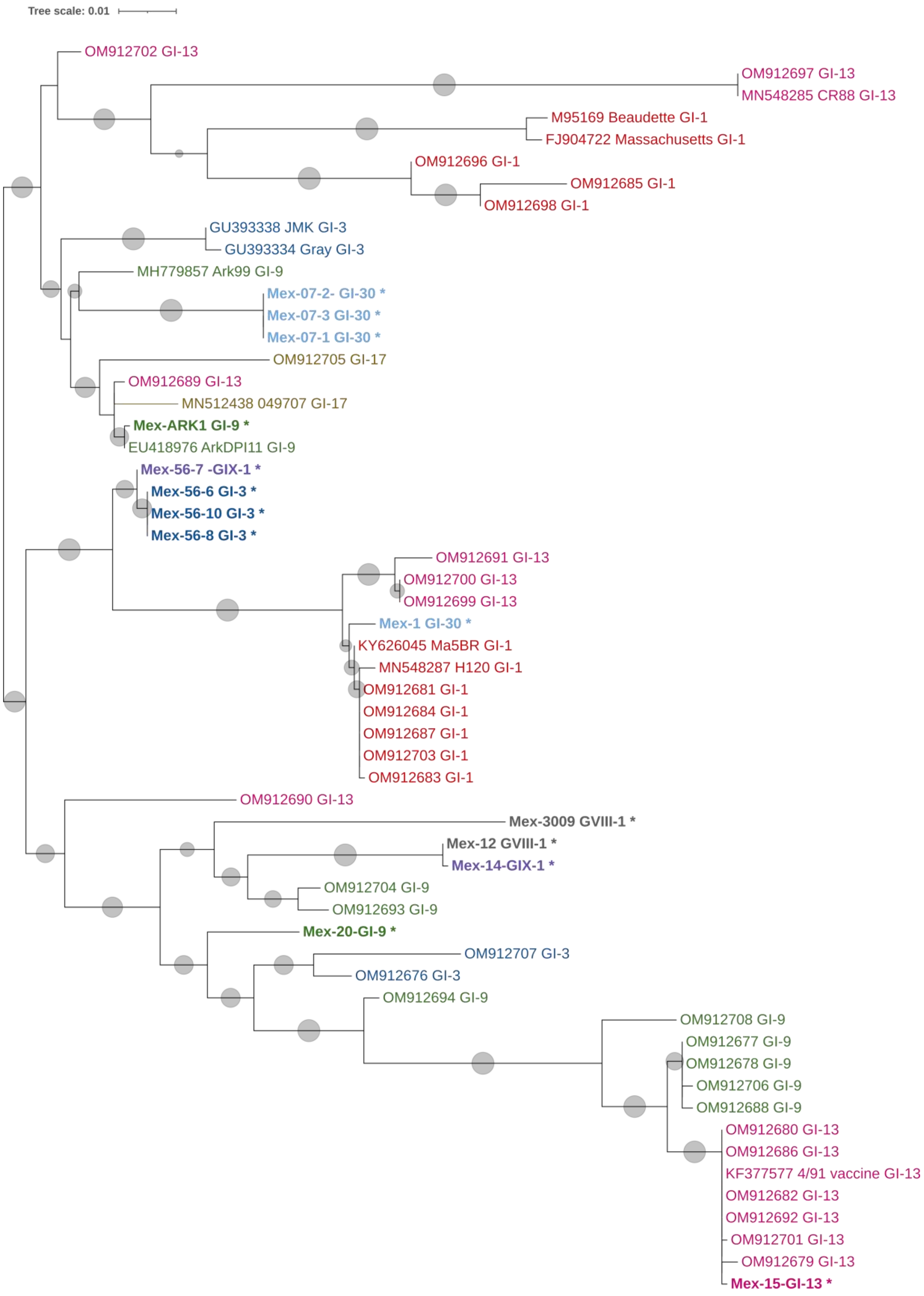

Figure S7. The phylogenetic tree was obtained with the maximum-likelihood method and GTR model with gamma distribution and invariant sites. Phylogenetic reconstruction was carried out using gene 7 sequences of the genomes here obtained (n = 14), other Mexican genomes available (n = 33), and reference or vaccine strains reported from Mexico (n = 10).
